# Serum Reactive Oxygen Metabolites as a Predictor of Clinical Disease Activity Index, Simplified Disease Activity Index, and Boolean Remissions in Rheumatoid Arthritis Patients Treated With Biologic Agents

**DOI:** 10.7759/cureus.19759

**Published:** 2021-11-19

**Authors:** Arata Nakajima, Keiichiro Terayama, Masato Sonobe, Yorikazu Akatsu, Junya Saito, Masaki Norimoto, Shinji Taniguchi, Ayako Kubota, Yasuchika Aoki, Koichi Nakagawa

**Affiliations:** 1 Orthopaedics and Rehabilitation, Toho University Sakura Medical Center, Sakura, JPN; 2 Rehabilitation Medicine, Toho University Sakura Medical Center, Sakura, JPN; 3 Orthopaedic Surgery, Toho University Sakura Medical Center, Sakura, JPN; 4 Orthopaedic Surgery, Toho University Omori Medical Center, Tokyo, JPN; 5 Orthopaedic Surgery, Eastern Chiba Medical Center, Togane, JPN

**Keywords:** reactive oxygen metabolites, remission, predictor, rheumatoid arthritis, biologic agents

## Abstract

Introduction

Reactive oxygen metabolites (ROMs) are metabolite hydroperoxides in the blood, and their serum levels were associated with the disease activity score 28 (DAS28) in patients with rheumatoid arthritis (RA). In this study, we aimed to investigate whether ROMs would be predictive of the clinical disease activity index (CDAI) remission, simplified disease activity index (SDAI) remission, or Boolean remission.

Materials and methods

Fifty-one biologic agents (BA)-naïve RA patients were included in this observational study. Associations between ROMs, C-reactive protein, matrix metalloproteinase-3, DAS28-erythrocyte sedimentation rate (ESR), CDAI, SDAI, and health assessment questionnaire (HAQ) at 12 weeks and the DAS28, CDAI, SDAI, and Boolean remission rates at 52 weeks were investigated.

Results

The DAS28, CDAI, SDAI, and Boolean remission rates at 52 weeks were 66.7, 52.9, 54.9, and 54.9%, respectively. A multivariate logistic regression analysis revealed that ROMs and HAQ at 12 weeks were associated with the CDAI, SDAI, and Boolean remission at 52 weeks. Receiver operating characteristic analyses demonstrated that the cut-off value for CDAI, SDAI, and Boolean remission was 389.5 U.Carr.

Conclusion

Reactive oxygen metabolites at 12 weeks of initial treatment with BAs was a predictor for CDAI, SDAI, and Boolean remission at 52 weeks. Serum levels of ROMs may be a useful biomarker in the current treatment strategy aiming at early remission of RA.

## Introduction

Rheumatoid arthritis (RA) is an autoimmune disease that induces synovial proliferation, leading to the development of joint destruction [1]. Oxidative stress induced by reactive oxygen species is thought to be one of the key mechanisms that underlie joint destruction and synovial proliferation observed in RA [2-5]. Excessive production of reactive oxygen species induces oxidative stress and damages proteins, lipids, and nucleic acids. Abundant amounts of reactive oxygen species have been detected in the synovial fluid of inflamed joints in patients with RA [6], and they play a role as one of the intracellular signaling molecules that amplify the synovial inflammation and proliferation [3]. Based on these observations, reactive oxygen species are thought to be involved in the pathophysiology of RA, but their clinical significance in the treatment of RA remains unknown.

Recently, a method of measuring reactive oxygen metabolites (ROMs) in blood, called the d-ROM test, has been developed. This method uses the Free Radical Analytical System 4 (FRAS 4, Wismarl, Italy) [7, 8]. Using this method, we previously showed that ROMs serum levels were associated with C-reactive protein (CRP) and the disease activity score (DAS) based on the examination of 28 joints (DAS28) in patients with RA [9]; however, their clinical significance as a biomarker during treatment of RA has not been elucidated fully.

To date, several factors have been reported to be associated with clinical remission during treatment [10-12]. Furthermore, novel biomarkers for predicting remission were identified [13, 14]; however, their clinical significance in the treatment of RA remains unclear. Based on these perspectives, we hypothesized that measurement of ROMs serum levels might be useful for checking current disease activity in the current treat-to-target strategy for RA. In this study, we investigated changes in ROMs during treatment with biologic agents (BAs) in patients classified as in-remission and non-remission at 52 weeks and verified whether ROMs could predict the future remission by clinical disease activity index (CDAI), simplified disease activity index (SDAI), and Boolean standards that are stricter remission standards than DAS28 [15, 16].

This article was previously published on a preprint server as Research Article: Terayama K, Sonobe M, Akatsu Y, et al. Clinical Significance of Serum Levels of ROM (Reactive Oxygen Metabolites) in Patients With Rheumatoid Arthritis Treated With Biologic Agents as a Predictor for the CDAI-, SDAI-, and Boolean-Remission. DOI: 10.21203/rs.3.rs-640054/v1. https://www.researchsquare.com/article/rs-640054/v1

## Materials and methods

Patients and background characteristics

In this observational study, 51 biologic agents (BA)-naïve patients with RA (mean age, 61.0 ± 13.6 y; disease duration, 7.24 ± 11.0 y) were evaluated for ROMs in addition to routine examinations. From patients who started treatment with tumor necrosis factor (TNF) inhibitors (infliximab, etanercept, adalimumab, golimumab, or certolizumab pegol) or tocilizumab from October 2011 to June 2016, blood samples were collected before administration of BAs and again at 4, 12, 24, and 52 weeks after initiation of administration. During this time, 46 patients had received one of the BAs listed above and had not been switched to other BAs.

The background characteristics of patients included in this study are shown in Table 1. The 51 patients with RA were classified as follows: high disease activity (n = 18), moderate disease activity (n = 29), low disease activity (n = 3), or in remission (n = 1). Overall, 88.2% of patients were treated with methotrexate (MTX, 8.78 ± 1.47 mg/week) and 68.6% with prednisolone (PSL, 5.54 ± 2.48 mg/day). Of the 51 patients, 22 received tocilizumab, and the others received TNF inhibitors (golimumab, 12; etanercept, 7; adalimumab, 4; certolizumab pegol, 4; and infliximab, 2). Five patients were switched to other BAs due to inadequate responses to their first BA: from golimumab to abatacept at 16 and 63 weeks (n = 2), from golimumab to tocilizumab at 12 and 35 weeks (n = 2), and from tocilizumab to golimumab at 24 weeks (n = 1).

**Table 1:** Patients’ demographic and disease characteristics at baseline BMI: body mass index, RF: rheumatoid factor, ROMs: reactive oxygen metabolites, CRP: C-reactive protein, MMP-3: matrix metalloproteinase-3, DAS28: 28-joint disease activity score, ESR: erythrocyte sedimentation rate, SDAI: simplified disease activity index, CDAI: clinical disease activity index, TJC: tender joint count, SJC: swollen joint count, PTGA: patient’s global assessment, MDGA: medical doctor’s global assessment, HAQ: health assessment questionnaire, PSL: prednisolone, MTX: methotrexate.

Number of patients	51
Age, years (range)	61.0 ± 13.6 (21-80)
Height, cm	157.1 ± 6.8
Body weight, kg	55.9 ± 9.4
BMI, kg/m^2^	22.6 ± 3.6
Diabetes, yes/no	9/42
Disease duration, years (range)	7.24 ± 11.0 (1-52)
RF-positive (%)	72.5
ROMs, U.Carr (range)	556 ± 123 (308-860)
CRP, mg/dL	2.82 ± 3.09
MMP-3, ng/mL	397 ± 354
DAS28-ESR	4.79 ± 1.26
CDAI	18.46 ± 12.74
SDAI	21.26 ± 14.21
TJC	5.6 ± 5.9
SJC	4.2 ± 5.1
PTGA, mm	45.1 ± 24.2
MDGA, mm	45.5 ± 21.9
HAQ	0.791 ± 0.610
PSL, mg/day (% usage)	5.54 ± 2.48 (68.6)
MTX, mg/week (% usage)	8.78 ± 1.47 (88.2)
Biologic agents	
Tocilizumab	22
Golimumab	12
Etanercept	7
Adalimumab	4
Certolizumab pegol	4
Infliximab	2

Venous blood samples were collected and analyzed for serum CRP and matrix metalloproteinase-3 (MMP-3). In our hospital, the normal reference value for CRP is 0.3 mg/dL. ROMs were also measured as described below.

For the assessment of RA disease activity, measurements of DAS28-ESR, CDAI, and SDAI were obtained during the same visit at which the blood samples were collected. Remission based on the DAS28, CDAI, and SDAI were defined as when the scores were ≤2.6, ≤2.8, and ≤ 3.3, respectively. Whether Boolean remission was achieved or not was also checked. RA patients also completed the health assessment questionnaire (HAQ). Approval for the study was received from the Institutional Review Board at Toho University Sakura Medical Center, and all patients gave their written consent to participate in this study. All activities were performed in accordance with the ethical standards set forth in the Declaration of Helsinki.

Measurement of oxidative stress markers in serum

To measure ROMs, the d-ROM test was performed using the Free Radical Analytical System 4 (FRAS 4) analyzer in accordance with the manufacturer’s analytical procedures. The details were described in our previous publications [9]. Reference values indicated by the FRAS 4 manufacturer range up to 300 U.Carr; values >300 U.Carr suggest the presence of oxidative stress [7, 8].

Statistical analyses

Results are expressed as mean ± standard deviation (SD). All laboratory data, DAS28, and HAQ scores were analyzed by the Last Observation Carried Forward (LOCF) method. Between-group differences were assessed by the Mann-Whitney U-test or the Steel-Dwass method. A multivariate logistic regression analysis was performed by the stepwise method to compute the odds ratios (ORs) and 95% confidence intervals (95% CIs) for the achievement of remission at 52 weeks. All statistical analyses were performed using SPSS (ver. 19) software (IBM Corp, Armonk, USA), and p-values < 0.05 were considered to indicate statistical significance.

## Results

Changes in DAS28-ESR, CDAI, SDAI, and remission rate during treatment

**Figure 1:**
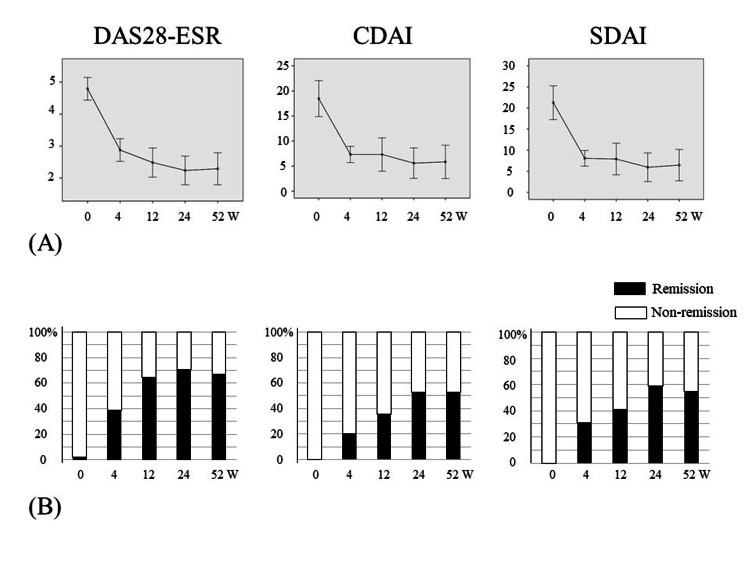
Changes in DAS28-ESR, CDAI, and SDAI, and the remission rate (A) Changes in DAS28-ESR, CDAI, and SDAI, and (B) the remission rate for all patients (n = 51) from baseline to 52 weeks. Error bars indicate ± SD. DAS28-ESR: disease activity score-28 with erythrocyte sedimentation rate; CDAI: clinical disease activity index; SDAI: simplified disease activity index; SD: standard deviation.

DAS28-ESR at baseline was 4.79 ± 1.26 and rapidly decreased to 2.87 ± 1.23 at 4 weeks. After 4 weeks, it gradually decreased to 2.29 ± 1.78 at 52 weeks (Fig. 1A). The remission rate increased from baseline and reached 66.7% at 52 weeks (Fig. 1B). CDAI at baseline was 18.5 ± 12.7 and rapidly decreased to 7.30 ± 5.60 at 4 weeks. After 4 weeks, it gradually decreased to 5.83 ± 11.9 at 52 weeks (Fig. 1A). The remission rate increased from baseline and reached 52.9% at 52 weeks (Fig. 1B). SDAI at baseline was 21.3 ± 14.2 and rapidly decreased to 8.07 ± 6.45 at 4 weeks. After 4 weeks, it gradually decreased to 6.45 ± 13.2 at 52 weeks (Fig. 1A). The remission rate increased from baseline and reached 54.9% at 52 weeks (Fig. 1B).

Changes in ROMs serum levels in the remission and non-remission groups at 52 weeks of treatment

**Figure 2:**
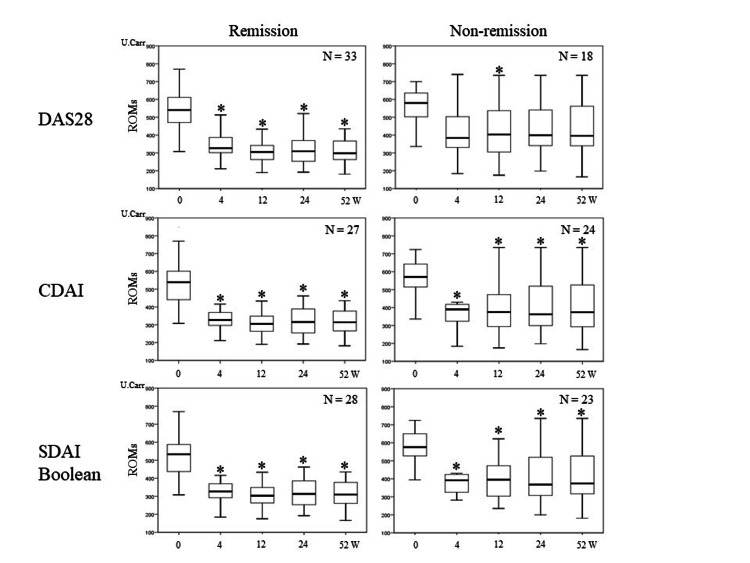
Changes in the serum level of ROMs in the 52-week remission and non-remission groups The distribution of patients with SDAI and Boolean remission was the same. Overall, the ROMs serum levels in both the remission and non-remission groups decreased between baseline and 4 weeks and remained low thereafter. In the remission group, there was a significant reduction in ROMs after 4 weeks compared to baseline levels (p < 0.05). In the non-remission group, a significant reduction was also seen after 4 weeks, but not at 4, 24, and 52 weeks based on the DAS28 standard. Thick horizontal line: median value; box: interquartile range (IQR); whiskers: most extreme points within 1.5-times the IQR from the limits of the box. Asterisks show significant differences compared to baseline values (*p < 0.05). ROMs: reactive oxygen metabolites; DAS28: 28-joint disease activity score; SDAI: simplified disease activity index, CDAI: clinical disease activity index.

As shown in Fig. 2, 33 patients achieved DAS28-remission at 52 weeks, while 18 did not. For CDAI remission, 27 patients achieved it, but 24 patients did not. The distribution of patients with SDAI and Boolean remission was the same: 28 patients achieved remission, but 23 patients did not. Overall, ROMs serum levels in both the remission and non-remission groups decreased between baseline and 4 weeks and remained low thereafter. In the remission group, there was a significant reduction in ROMs after 4 weeks compared to baseline levels (p < 0.05). In the non-remission group, a significant reduction was also seen after 4 weeks based on the CDAI, SDAI, and Boolean standards, but not at 4, 24, and 52 weeks based on the DAS28 standard.

Analyses to identify factors at 12 weeks of treatment that are associated with 52-week remission

Since temporal changes in ROMs in the remission and non-remission groups at 52 weeks followed different patterns after 12 weeks, we elected to analyze factors at 12 weeks that may be associated with 52-week remission. We performed a univariate analysis for variables including ROMs, CRP, MMP-3, DAS28, CDAI, SDAI, and HAQ at 12 weeks between the 52-week remission and non-remission groups. For DAS28 remission, ROMs, MMP-3, DAS28, CDAI, SDAI, and HAQ were significantly different. For CDAI, SDAI, and Boolean remission, ROMs, DAS28, CDAI, SDAI, and HAQ were significantly different (p < 0.05, Table 2).

**Table 2:** Comparison of the factors at 12 weeks between the remission and non-remission groups at 52 weeks ROMs: reactive oxygen metabolites, CRP: C-reactive protein; MMP-3: matrix metalloproteinase-3, DAS28: 28-joint disease activity score, CDAI: clinical disease activity index, ESR: erythrocyte sedimentation rate; SDAI: simplified disease activity index, HAQ: health assessment questionnaire. *Significantly different between groups (p< 0.05).

	DAS28				CDAI				SDAI & Boolean		
	Remission N = 33	Non-remission N = 18	p		Remission N = 27	Non-remission N = 24	p		Remission N = 28	Non-remission N = 23	p
ROMs (U.Carr)	306 ± 72.5	434 ± 131	0.001*		307 ± 61.4	396 ± 138	0.007*		302 ± 65.2	405 ± 132	0.002*
CRP (mg/dL)	0.052 ± 0.13	1.53 ± 2.98	0.057		0.087 ± 0.21	1.06 ± 2.59	0.079		0.084 ± 0.21	1.11 ± 2.64	0.077
MMP3 (ng/mL)	100 ± 73.4	198 ± 157	0.029*		105 ± 68.1	160 ± 147	0.103		105 ± 66.9	164 ± 150	0.090
DAS28-ESR	1.75 ± 0.900	3.95 ± 1.76	0.000*		1.86 ± 1.10	3.18 ± 1.83	0.004*		1.85 ± 1.08	3.26 ± 1.84	0.003*
CDAI	3.87 ± 4.62	14.2 ± 17.8	0.031*		3.86 ± 5.05	11.2 ± 15.7	0.037*		3.86 ± 4.96	11.5 ± 16.0	0.036*
SDAI	3.99 ± 4.68	15.7 ± 20.3	0.031*		4.03 ± 5.14	12.3 ± 17.9	0.039*		4.03 ± 5.05	12.6 ± 18.2	0.038*
HAQ	0.254 ± 0.353	0.824 ± 0.754	0.008*		0.212 ± 0.318	0.703 ± 0.697	0.003*		0.245 ± 0.358	0.685 ± 0.707	0.011*

A multivariate logistic regression analysis revealed the following: (1) DAS28 was associated with the DAS28-remission (OR: 0.172, 95% CI: 0.063-0.467, p = 0.001); (2) ROMs and HAQ were associated with the CDAI-remission (OR: 0.991; 95% CI: 0.983-0.999, p = 0.029 for ROM; OR: 0.102, 95% CI: 0.017-0.605, p = 0.012 for HAQ); and (3) ROMs and HAQ were also associated with the SDAI- and Boolean-remission (OR: 0.989, 95% CI: 0.980-0.997, p = 0.011 for ROM; OR: 0.166, 95% CI: 0.031-0.889, p = 0.036 for HAQ) (Table 3).

**Table 3:** A multivariate logistic regression analysis determining factors at 12 weeks of treatment associated with the 52-week remission ROMs: reactive oxygen metabolites, DAS28: 28-joint disease activity score, ESR: erythrocyte sedimentation rate; CDAI: clinical disease activity index, SDAI: simplified disease activity index, HAQ: health assessment questionnaire, CI: confidence interval. *Significant differences according to a logistic regression analysis (p< 0.05).

	DAS28				CDAI				SDAI & Boolean		
	Odds ratio	95% CI	p		Odds ratio	95% CI	p		Odds ratio	95% CI	p
ROMs	0.992	0.976-1.007	0.056		0.991	0.983-0.999	0.029*		0.989	0.980-0.997	0.011*
DAS28-ESR	0.172	0.063-0.467	0.001*		0.879	0.31-2.497	0.774		0.91	0.320-2.585	0.859
CDAI	0.85	0.041-17.57	0.228		0.581	0.063-5.339	0.782		0.551	0.054-5.683	0.617
SDAI	1.326	0.062-28.25	0.232		1.722	0.185-16.013	0.800		1.74	0.167-18.108	0.643
HAQ	0.027	0.001-1.053	0.222		0.102	0.017-0.605	0.012*		0.166	0.031-0.889	0.036*

**Figure 3:**
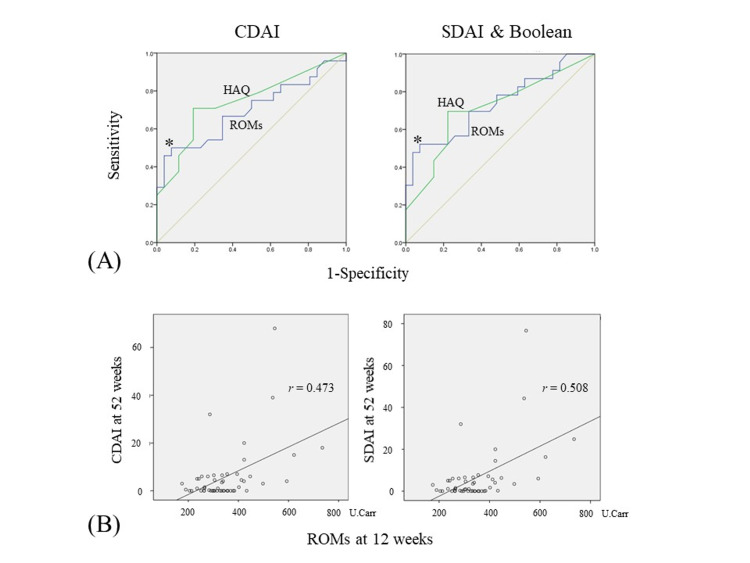
ROC curves for ROMs and HAQ, and correlation between ROMs and CDAI or SDAI remission (A) ROC curves for ROMs (blue line) and HAQ (green line) at 12 weeks predicting the CDAI, SDAI, and Boolean remission at 52 weeks. The cut-off value for ROMs corresponds to 389.5 U.Carr (indicated as an asterisk). (B) Correlation between ROMs at 12 weeks and CDAI or SDAI at 52 weeks. CDAI and SDAI at 52 weeks were correlated with ROMs at 12 weeks (r = 0.473, p < 0.01 for CDAI; r = 0.508, p < 0.01 for SDAI). ROC: receiver operating characteristic; ROMs: reactive oxygen metabolites; SDAI: simplified disease activity index, CDAI: clinical disease activity index

As shown in Fig. 3A, for CDAI remission, the area under the curve (AUC) of the receiver operating characteristic (ROC) curve for ROMs (blue line) was 0.696 (sensitivity: 50.0%, specificity: 92.3%). For the SDAI and Boolean remission, it was 0.737 (sensitivity: 52.2%, specificity: 92.6%). The cut-off value that discriminated remission from non-remission for CDAI, SDAI, and Boolean standards was determined to be 389.5 U.Carr.

We further analyzed the correlation between ROMs at 12 weeks and CDAI or SDAI at 52 weeks, and found that ROMs at 12 weeks were correlated with CDAI (r = 0.473, p < 0.01) and SDAI (r = 0.508, p < 0.01) (Fig. 3B). We also analyzed the correlation between ROMs at baseline and CDAI or SDAI at 52 weeks; however, no significant correlations were detected (data not shown).

## Discussion

In this study, we demonstrated that the ROMs serum level at 12 weeks in the treatment with BAs was a useful biomarker for predicting the 52-week remission for CDAI, SDAI, and Boolean standards. The HAQ at 12 weeks was also a predictor for the 52-week remission. The AUC values of ROMs for the CDAI-remission and SDAI or Boolean remission were 0.696 and 0.737, respectively, both of which were moderately accurate; those of HAQ were 0.743 and 0.712, respectively, which were almost equivalent to those of ROMs. The sensitivity for the CDAI remission and SDAI or Boolean remission were 50.0% and 52.2%, respectively, although their specificity was over 90%. Considering these results, at present, ROMs may not be a highly accurate predictor for the 52-week remission for CDAI, SDAI, and Boolean standards. However, CRP and MMP-3, which are biomarkers used to monitor disease activity and joint damage progression in routine clinical practice, were not identified as predictors for the 52-week remission in a logistic regression analysis. These results suggest that ROMs are at least superior to CRP and MMP-3 in terms of predicting future remission.

It seems important to determine the appropriate time point during treatment for predicting 52-week remission. In addition to 12 weeks, we investigated whether the ROMs serum level at baseline and at 4 weeks could predict 52-week remission in a logistic regression analysis, but we could not find significant factors (data not shown). Therefore, we decided that 12 weeks was the appropriate time point to measure ROMs serum levels for predicting 52-week remission. The finding that the ROMs level at 12 weeks can predict the 52-week remission for CDAI, SDAI, and Boolean standards could make ROMs a useful biomarker for monitoring current treatment strategies relatively early.

Previous studies have demonstrated that the baseline HAQ was associated with remission after treatment. Quintana-Duque et al showed that a lower HAQ-disability index (DI) score and absence of autoantibodies were predictive of remission [10]. Hoshi et al showed that the baseline Japanese version of HAQ was a negative predictor of Boolean-based remission in patients treated with tocilizumab [11]. Pomirleanu et al showed that initial DAS28-ESR, HAQ-DI, CRP, rheumatoid factor, and anti-citrullinated protein antibody were associated with an increased likelihood of remission and low disease activity [17]. These findings suggest that the baseline HAQ could be a useful parameter for predicting remission. Actually, in the present study, the HAQ and ROMs were identified as factors associated with the 52-week remission. However, the HAQ is in itself a parameter to check functional disabilities rather than a biomarker. From the viewpoint of biomarkers for monitoring treatment progress, ROMs seem to be superior to HAQ.

In this study, the distribution of patients with CDAI remission was the same as that of patients with SDAI and Boolean remission except for one patient. This leads to quite similar results, which are shown in Tables 2 and 3 and Figures 1-3, between the remission and non-remission groups for CDAI and SDAI or Boolean standards. Because we used tocilizumab for approximately 40% (22/51) of patients, the SDAI and Boolean standards, which are affected by CRP, might have been evaluated better. If we used TNF inhibitors for more patients, the distribution of patients for CDAI remission and SDAI or Boolean remission may be altered.

A predictive factor for DAS28 remission was DAS28 at 12 weeks; however, this is an expected result. The serum ROMs levels were not identified (p = 0.056). When DAS28 was excluded from independent variables, ROMs and HAQ were again identified (OR: 0.985, 95% CI: 0.975-0.995, p = 0.005 for ROMs; OR: 0.075, 95% CI: 0.010-0.570, p = 0.012 for HAQ, data not shown). DAS28 may differ among physicians and also is not a biomarker. Therefore, we believe that monitoring ROMs values allows physicians to predict future remission rather than monitoring DAS28.

The strong points of this study are that we demonstrated the superiority of ROMs to other clinical parameters used in routine clinical practice, such as CRP, MMP-3, DAS28, CDAI, and SDAI, as a predictor for the 52-week remission by CDAI, SDAI, and Boolean standards. Although the ROMs cut-off value that discriminates remission from non-remission does not have high sensitivity, controlling serum ROMs levels tightly may allow patients to achieve remission by CDAI, SDAI, and Boolean standards in the early stage during treatment with BAs.

This study has some limitations. First, the sample size was small, and background characteristics such as disease duration, PSL, and MTX doses, and concomitant diseases like diabetes varied among patients. Second, a variety of BAs, including TNF inhibitors and tocilizumab, were used. The effect of BAs on the inhibition of ROMs may be different among BAs. Third, the d-ROM test does not directly measure reactive oxygen and free radicals but rather quantifies the metabolite hydroperoxides** **to evaluate oxidative stress. Therefore, other factors/conditions may influence these measurements. Fourth, the association of ROMs with joint destruction has not been investigated. The relationship between serum ROMs levels and radiographic progression of joint damage should be analyzed in the future. Finally, although oxidative stress is involved in the pathophysiology of many diseases in humans [18-25], its role in the pathobiology of RA remains unclear. Further studies are required to establish the clinical significance of oxidative stress in RA.

## Conclusions

The ROMs serum level at 12 weeks after treatment initiation with BAs is a predictive factor of 52-week remission by CDAI, SDAI, and Boolean standards. The ROMs serum level may be a useful biomarker for achieving early remission in the current treatment strategy aiming at the early remission of RA.
